# Evaluations of rationally designed rift valley fever vaccine candidate RVax-1 in mosquito and rodent models

**DOI:** 10.1038/s41541-022-00536-3

**Published:** 2022-09-21

**Authors:** Tetsuro Ikegami, Eduardo Jurado-Cobena, Cigdem Alkan, Jennifer K. Smith, Lihong Zhang, Birte Kalveram, Terry L. Juelich, Allen T. Esterly, Jahnavi R. Bhaskar, Saravanan Thangamani, Alexander N. Freiberg

**Affiliations:** 1 Department of Pathology, The University of Texas Medical Branch at Galveston, 301 University Blvd, Galveston, TX 77555 USA; 2 The Sealy Institute for Vaccine Sciences, The University of Texas Medical Branch at Galveston, 301 University Blvd, Galveston, TX 77555 USA; 3 The Center for Biodefense and Emerging Infectious Diseases, The University of Texas Medical Branch at Galveston, 301 University Blvd, Galveston, TX 77555 USA; 4 Department of Microbiology and Immunology, The University of Texas Medical Branch at Galveston, 301 University Blvd, Galveston, TX 77555 USA; 5 Department of Microbiology and Immunology, SUNY Upstate Medical University, Syracuse, NY 13210 USA; 6 SUNY Center for Vector-Borne Diseases, SUNY Upstate Medical University, Syracuse, NY 13210 USA; 7 Institute for Global Health and Translational Science, SUNY Upstate Medical University, Syracuse, NY 13210 USA

**Keywords:** Live attenuated vaccines, Virus-host interactions

## Abstract

Rift Valley fever (RVF) is a mosquito-borne zoonosis endemic to Africa and the Arabian Peninsula, which causes large outbreaks among humans and ruminants. Single dose vaccinations using live-attenuated RVF virus (RVFV) support effective prevention of viral spread in endemic countries. Due to the segmented nature of RVFV genomic RNA, segments of vaccine strain-derived genomic RNA could be incorporated into wild-type RVFV within co-infected mosquitoes or animals. Rationally designed vaccine candidate RVax-1 displays protective epitopes fully identical to the previously characterized MP-12 vaccine. Additionally, all genome segments of RVax-1 contribute to the attenuation phenotype, which prevents the formation of pathogenic reassortant strains. This study demonstrated that RVax-1 cannot replicate efficiently in orally fed *Aedes aegypti* mosquitoes, while retaining strong immunogenicity and protective efficacy in an inbred mouse model, which were indistinguishable from the MP-12 vaccine. These findings support further development of RVax-1 as the next generation MP-12-based vaccine for prevention of Rift Valley fever in humans and animals.

## Introduction

Rift Valley fever (RVF) is a mosquito-borne zoonotic viral disease endemic to sub-Saharan Africa, Egypt, Madagascar, the Comoros, Saudi Arabia, and Yemen, which is caused by the Rift Valley fever virus (RVFV: genus *Phlebovirus*, family *Phenuiviridae*)^1–3^. In nature, RVFV is spread via vertical viral transmission in floodwater *Aedes* mosquitoes, and horizontal viral transmission among various species of mosquitoes and susceptible animals. RVF outbreaks are typically preceded by high rates of spontaneous abortion and fetal malformation in sheep, cattle, and goats, and lethal hepatitis in newborn lambs and goat kids^3^. RVFV infection in humans occurs via direct contact with bodily fluids of infected animals or through the bite of infected mosquitoes. Clinically, RVF patients show a biphasic febrile illness with complications occurring in less than 8% of patients, which include hemorrhagic fever, encephalitis, or retinitis, with 0.5 to 1.0% overall mortality rate^4–6^. RVFV is a Risk Group 3 pathogen, classified as a Category A Priority Pathogen by the NIAID/NIH in the United States (U.S.), an Overlap Select Agent by the U.S. Department of Health and Human Services (HHS) and Agriculture (USDA), is on the R&D Blueprint list of priority diseases from the World Health Organization (WHO), and is a notifiable disease to the World Organization for Animal Health (OIE). RVFV has a tripartite RNA genome comprised of sections L, M and S, which encodes four structural proteins (N, Gn, Gc, and L) and three non-structural proteins (NSs, NSm, and 78kD). The NSs protein serves as a major virulence factor for RVFV^7,8^, while 78kD and NSm proteins play roles in viral dissemination in mosquitoes and suppression of apoptosis in mammalian cells, respectively^9–11^.

In the U.S., the live-attenuated MP-12 candidate vaccine was developed via serial passage of pathogenic strain ZH548 in the presence of chemical mutagen 5-fluorouracil^12^. The MP-12 candidate vaccine has been studied for the purpose of preventing both human and animal RVF. MP-12 has been tested in phase 1 and 2 clinical trials for safety and immunogenicity in healthy adults^13,14^, and has been conditionally licensed for veterinary use in the U.S.^15^. The attenuation of the MP-12 vaccine strain is largely derived from the combination of two amino acid changes (Gn-Y259H and Gc-R1182G) in the M segment. These are combined with mutations in the L segment (L-V172A, L-M1244I) to produce a temperature sensitive (TS) phenotype which limits viral replication above 38 °C^16–18^. Based on the demonstrated immunogenicity in humans and ruminants, which lasts for at least several years with a single dose^14,19,20^, vaccination with MP-12 might provide an excellent tool for controlling outbreaks. Campbell et al. (2021), however, showed that mosquito vectors (*Ae. aegypti* or *Culex tarsalis*) fed with MP-12 had efficient viral dissemination by 14 days post infection (dpi)^21^. In endemic countries, animal vaccination can be performed using automatic syringes, which may increase the risk of mixing the vaccine and wild-type RVFV strains via needle contamination with viremic blood^22^. The Target Product Profile drafted by the WHO showed concern for the potential creation of reassortant strains via the use of live-attenuated vaccines in animals or humans^23^.

In this study, we aimed to generate and characterize a next generation recombinant MP-12 strain designated as RVax-1. RVax-1 genome RNA encodes 566 silent mutations, which confers attenuation via S, M, or L segment upon the formation of reassortant strains with wild-type L, M, or S segments^24^. RVax-1 M-segment lacks 78kD and NSm genes, which can minimize the dissemination of RVax-1 in mosquito vectors. Here, we describe the rescue of an infectious clone of RVax-1 from cloned cDNA in Vero cells, the genetic stability of the 566 individual silent mutations of RVax-1, viral dissemination capability in mosquitoes, and immunogenicity and protective efficacy of RVax-1 in the inbred C57BL/6 mouse model. The Proof-of-Concept for the rationally designed RVax-1 candidate vaccine will support further preclinical and clinical developments.

## Results

### Rescue of infectious clone of RVax-1 from cloned cDNA in Vero cells

**Fig. 1:**
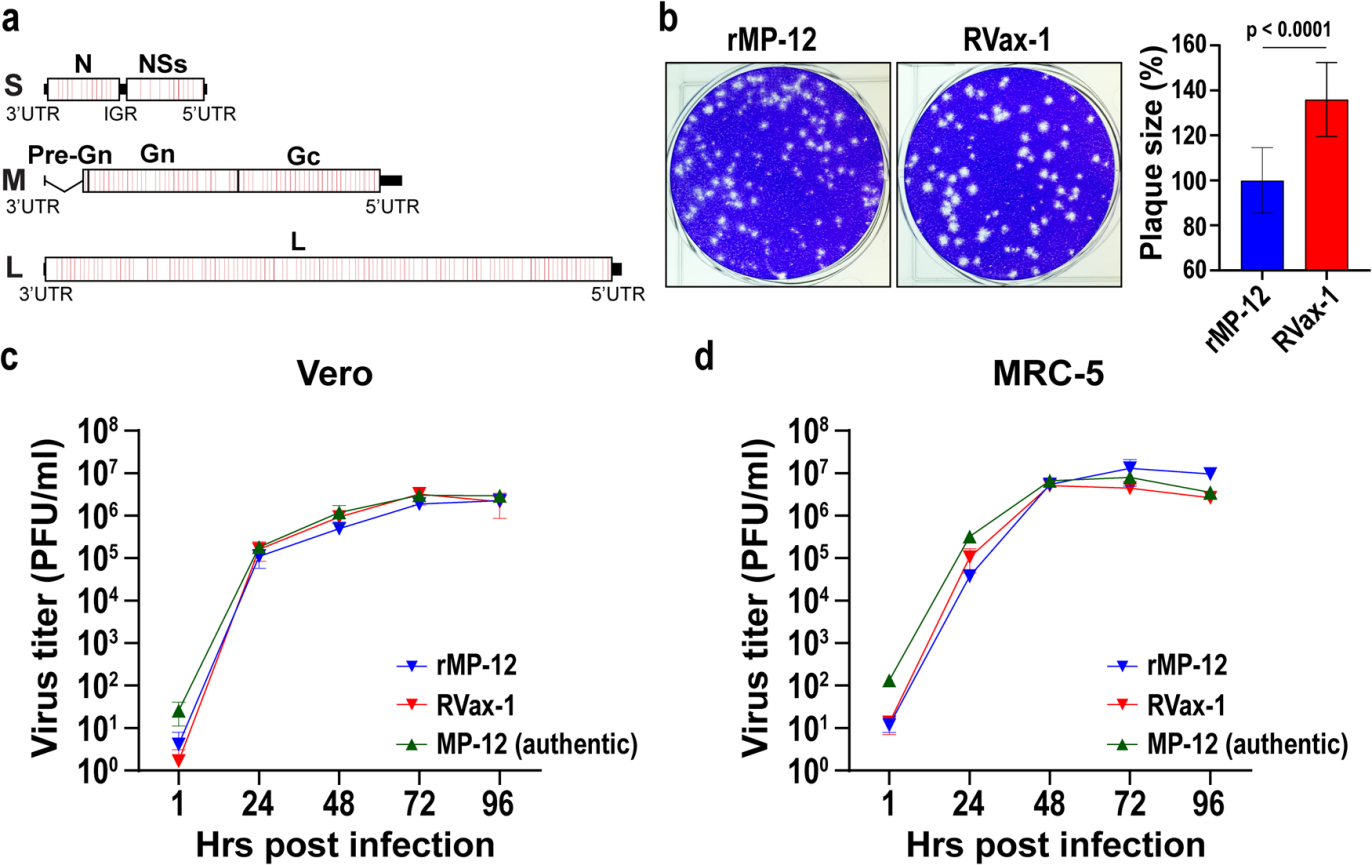
Genetic structure and growth kinetics of RVax-1 in Vero or MRC-5 cells. **a** Schematic representation of the RVax-1 S, M, and L segments, which encode a deletion at nt. 21–384 in the M-segment, and 566 silent mutations throughout the N, M, and L ORFs. Locations of individual silent mutations are labeled in red lines, which are found as a cluster every 50 nt. **b** Plaque phenotypes of rMP-12 and RVax-1 in Vero cells at 4 dpi. The graph represents the means ± standard deviations of relative diameter lengths from 20 randomly selected plaques. Unpaired *t*-test was used for the comparison of two groups (*t* = 7.319, df = 38). Vero cells (**c**) or MRC-5 cells (**d**) were infected with MP-12 vaccine lot-7-2-88, rMP-12 or RVax-1 in Vero cells at MOI 0.01. Virus replication kinetics at 35 °C are shown. Means ± standard deviations of triplicate wells are shown.

We designed the RVax-1 vaccine strain as shown in Fig. 1a. RVax-1 encodes 566 silent mutations in the MP-12 backbone: i.e., 36, 37, 167, 326 mutations in N, NSs, M, and L open reading frames (ORFs), respectively. The pattern of silent mutations was designed to be identical to that of the rMP12-GM50 strain, in which a few silent mutations are located at every 50 nt in N, NSs, M, and L ORFs^24^. In addition, RVax-1 encodes an in-frame deletion (∆21/381) in the M-segment. Infectious clones of recombinant MP-12 (rMP-12) or RVax-1 were rescued from cloned cDNA directly from Vero cells (*Chlorocebus* sp., kidney epithelial cells, ATCC CCL-81) using a simian RNA polymerase I promoter-driven reverse genetics system^25^. Both rMP-12 and RVax-1 formed clear plaques in Vero cells, whereas the mean plaque size of RVax-1 was approximately 1.4 times larger than that of rMP-12 at 4 dpi (Fig. 1b). RVax-1, which encodes a deletion of NSm/78kD genes, induced early apoptotic cell death in Vero cells, while parental rMP-12 did not (Supplementary Figure 1). RVax-1, rMP-12, or authentic MP-12 Lot 7-2-88 showed similar replication kinetics in Vero cells or MRC-5 cells (human lung diploid cells, ATCC CCL-171), both of which are potential RVF vaccine substrates, at 35 °C (Multiplicity of infection, MOI: 0.01) (Fig. 1c, d). To minimize the potential occurrence of reversion mutations for the TS mutations derived from the MP-12-backbone^16,17^, we maintained the temperature of culture cells at 35 °C for amplification of stock viruses.

### Genetic stability of 566 silent mutations in RVax-1

The genetic stability of the 566 silent mutations in RVax-1 was analyzed during ten serial passages in Vero cells at 35 °C. Culture supernatants of RVax-1-infected Vero cells were blindly passaged ten times. Subsequent back-titrations of culture supernatants determined the MOI of each passage, while Northern blot analysis of P2, P5, or P10 RNA from three independent passage experiments showed that there were no detectable defective interfering RNAs (Supplementary Fig. 2). Subsequently, genetic variants of P5 and P10 RNA samples were analyzed by RNA-Seq. For the 566 silent mutations, genetic changes above 0.1% of the total populations were characterized (Table 1). None of the 566 mutation sites had genetic subpopulations above 0.6%. Genetic subpopulations for the 25 loci showed (i) reversion to MP-12 sequences (15 sites), (ii) silent mutations to non-MP-12 sequences (6 sites), or (iii) amino acid changes (4 sites). One location (S segment: U1207C) had a slight accumulation of mutant population from P5 (0.2%) to P10 (0.6%) in one of the three experiments, whereas no other loci was detected to have the same genetic subpopulation at both P5 and P10. At 2 locations (M segment: A2386G, and L segment C2850U) the same genetic subpopulation emerged in two of the three experiments.

**Table 1:** Genetic subpopulation changes among 566 RVax-1 silent mutations during serial virus passages in Vero cells.

Gene	Mutations	Exp-1 P5/P10	Exp-2 P5/P10	Exp-3 P5/P10	Note
N	C293U	0.25^a^/−	−/−	−/−	Silent mutation (MP-12 sequence)
N	U350A	−/−	−/−	−/0.19	Silent mutation (MP-12 sequence)
N	C542U	−/−	−/0.24	−/−	Silent mutation (MP-12 sequence)
N	A546G	−/−	−/−	0.34/−	Amino acid change (S to G)
NSs	C904G	−/−	−/0.12	−/−	Amino acid change (E to D)
NSs	A1156G	−/−	−/−	−/0.29	Silent mutation (non-MP-12 sequence)
NSs	A1204G	−/−	0.17/−	−/−	Silent mutation (MP-12 sequence)
NSs	U1207C	0.2/0.6	−/−	−/−	Silent mutation (MP-12 sequence)
NSs	A1252U	−/−	0.24/−	−/−	Amino acid change (D to E)
NSs	G1350A	0.43/−	−/−	−/−	Amino acid change (R to C)
Gc	U1891A	−/−	−/−	−/0.15	Silent mutation (MP-12 sequence)
Gc	C2236U	−/−	−/−	−/0.38	Silent mutation (MP-12 sequence)
Gc	A2386G	−/−	−/0.47	−/0.53	Silent mutation (MP-12 sequence)
Gc	U3139C	−/0.25	−/−	−/−	Silent mutation (MP-12 sequence)
L	A804G	−/−	−/−	−/0.24	Silent mutation (MP-12 sequence)
L	G1791A	−/−	−/0.45	−/−	Silent mutation (MP-12 sequence)
L	A2799G	−/−	−/−	−/0.33	Silent mutation (MP-12 sequence)
L	C2850U	0.36/−	−/0.38	−/−	Silent mutation (non-MP-12 sequence)
L	C3060G	−/0.27	−/−	−/−	Silent mutation (non-MP-12 sequence)
L	C3696U	−/−	−/−	−/0.42	Silent mutation (MP-12 sequence)
L	U3858G	−/−	−/0.11	−/−	Silent mutation (MP-12 sequence)
L	U4251C	−/−	−/−	−/0.15	Silent mutation (non-MP-12 sequence)
L	U4911C	−/0.44	−/−	−/−	Silent mutation (MP-12 sequence)
L	G5199A	−/0.39	−/−	−/−	Silent mutation (non-MP-12 sequence)
L	G5754A	−/−	−/0.34	−/−	Silent mutation (non-MP-12 sequence)

^a^The numbers represent percentages of viral subpopulations emerged at indicated mutation sites of RVax-1.

### Dissemination capability of RVax-1 in mosquitoes

**Fig. 2:**
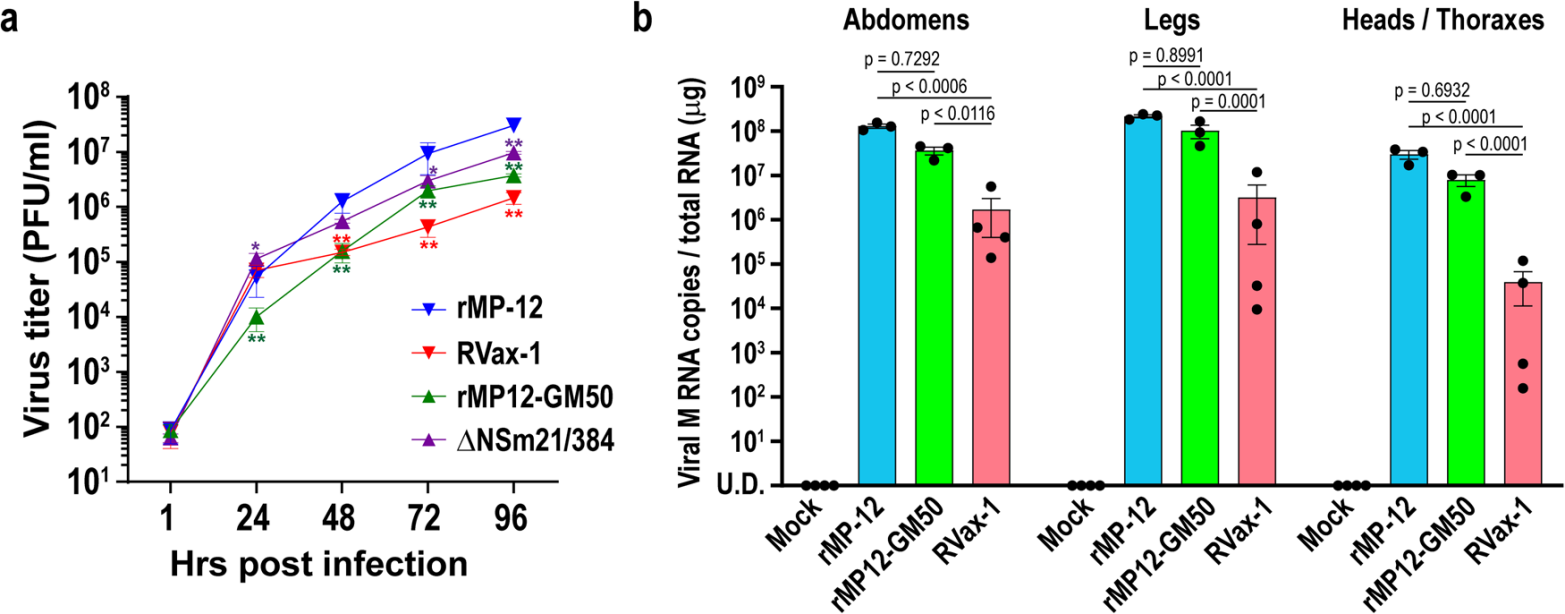
Characterization of RVax-1 replication in mosquitoes. **a** C6/36 cells were infected with rMP-12, RVax-1, rMP12-GM50, or rMP12-∆NSm21/384 at MOI 0.01. Virus replication kinetics at 28 °C are shown. The graph represents the means ± standard deviations of triplicate wells (two-way ANOVA; *p < 0.05, **p < 0.01, vs. rMP-12, *F* = 13.47, df = 12). **b** *Aedes aegypti* were fed on blood meals containing medium (mock), rMP-12, rMP12-GM50, or RVax-1. Total RNA from abdomen, leg, or head/thorax/wing from three pools (*n* = 10 per pool) of mosquitoes at 14 dpi in each group were used for qRT-PCR analysis. Copy number of viral M segment RNA was measured by Taqman qRT-PCR using the standard curve for in vitro synthesized RVFV M segment RNA. Means ± standard errors of three different pools are shown in a scattered dot plot graph (two-way ANOVA: *F* = 2.178, df = 6).

Kreher et al. (2014) showed that NSm proteins support efficient virus replication in *Ae. albopictus* C6/36 cells, whereas 78kD proteins have a role in viral replication in *Ae. aegypti* mosquitoes^9^. Deletions of both 78kD and NSm genes also affected the dissemination of the RVFV ZH501 strain in *Ae. aegypti* and *Cx. tarsalis* mosquitoes^11,21^. To characterize RVax-1 replication in mosquito cells, we determined its viral replication kinetics in C6/36 cells. These cells were infected with rMP-12, rMP12-GM50 (584 silent mutations, intact 78kD and NSm genes)^24^, arMP12-∆NSm21/384 (deletion of 78kD and NSm genes)^26^, or RVax-1 (566 silent mutations and deletion of 78kD and NSm genes) at MOI 0.01. Replication kinetics at 28 °C are shown in Fig. 2a. The rMP12-GM50 replicated 5–8-fold less efficiently compared to parental rMP-12 between 24 and 96 h post infection (hpi). The arMP12-∆NSm21/384 replicated efficiently at 24 hpi, whereas the titers were decreased by 2–3-fold between 48 and 96 hpi compared to rMP-12 titers. RVax-1 replicated efficiently at 24 hpi, and then decreased the titer by 8–20-fold between 48 and 96 hpi.

To test the dissemination capability of RVax-1 in mosquitoes, *Ae. aegypti* mosquitoes (3–5-day old) were fed on sheep blood spiked with Dulbecco’s modified Eagle medium (DMEM) (mock-infected control), or concentrated viruses (rMP-12, rMP12-GM50, or RVax-1). Back titration of blood meals measured actual virus concentrations as 1.7 × 10^8^ plaque forming units (PFU) for rMP-12, 1.0 × 10^8^ PFU for rMP12-GM50, and 1.7 × 10^8^ PFU for RVax-1. At 14 days post engorgement, abdomen, leg, and head/thorax/wing were collected from live mosquitoes for total RNA extraction (three replicates, *n* = 10 per replicate). The qRT-PCR analysis revealed that rMP12-GM50 M segment RNA copies were 3.6-, 2.1-, or 3.8-fold less than those of rMP-12 in abdomen, leg, or head/thorax/wing, respectively (no statistical significance), whereas RVax-1 M segment RNA copies were 76.2-, 67.9-, or 759.0-fold less than those of rMP-12 in abdomen, leg, or head/thorax/wing, respectively (Two-way analysis of variance; ANOVA, *p* < 0.01) (Fig. 2b).

**Fig. 3:**
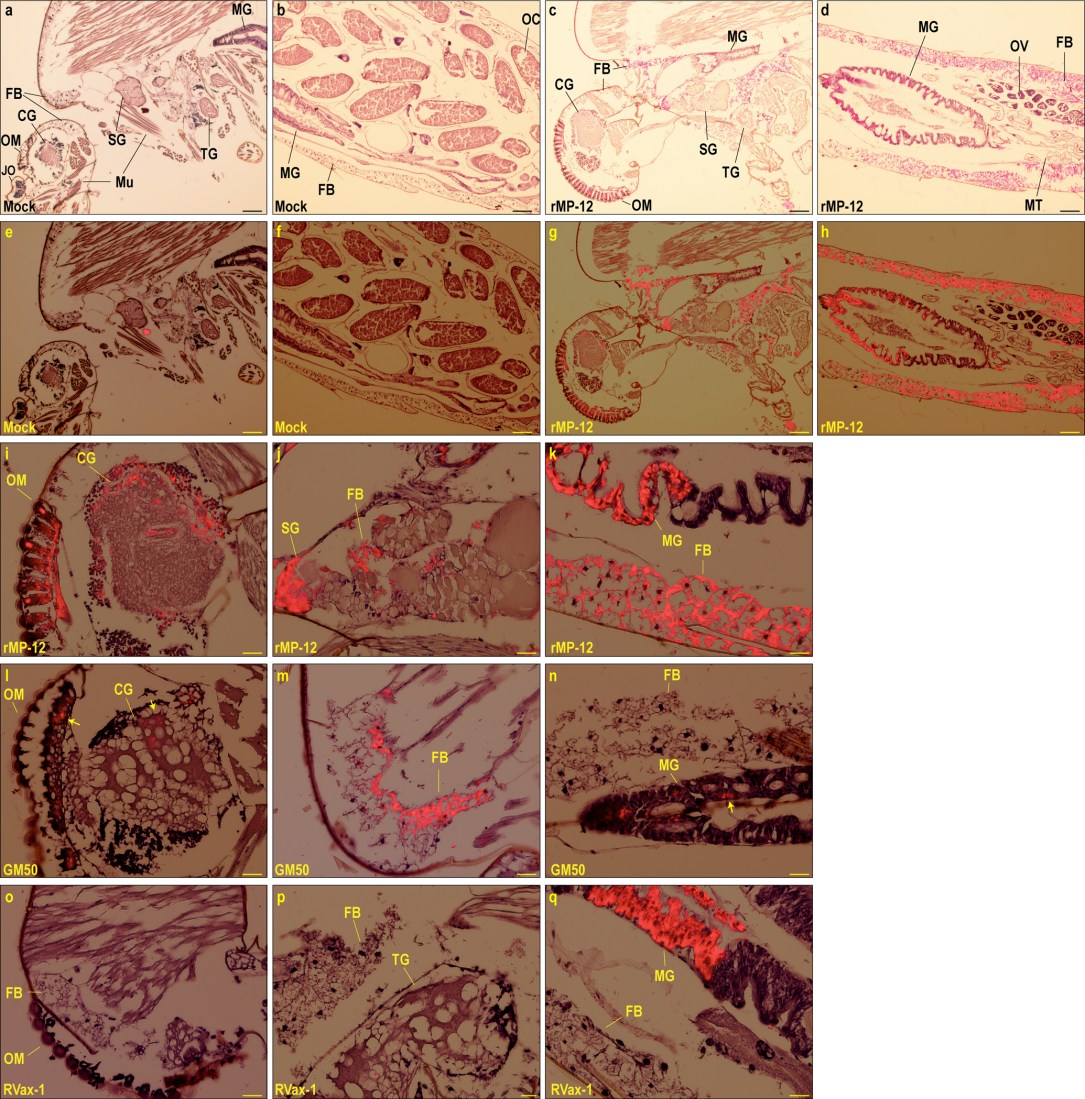
Viral antigen distributions in Aedes aegypti infected with rMP-12, rMP12-GM50, or RVax-1. *Aedes aegypti* were fed on blood meals containing medium (mock), rMP-12, rMP12-GM50, or RVax-1. Whole bodies were fixed with 10% neutral buffered formalin at 14 dpi. Immunohistochemistry using anti-RVFV N antibody are shown. Low magnification images are from mock-infected mosquitoes (**a**, **b**, **e**, **f**) or rMP-12-infected mosquitoes (**b**, **d**, **g**, **h**) under brightfield microscopy (**a**–**d**) or merged with TRITC fluorescent image (**e**–**h**). Positive signals are shown in magenta (brightfield) or red (TRITC). Bars represent 100 µm. High magnification brightfield images from rMP-12-infected (**i**–**k**), rMP12-GM50-infected (**l**–**n**), or RVax-1-infected mosquitoes (**o**–**q**) in head, thorax, or abdomen are shown as merged with TRITC fluorescent images, respectively. Bars represent 25 µm. Arrows in L and N images indicate RVFV N antigens. FB fat body, MG midgut, MT Malpighian tubules, SG salivary gland, Mu muscle, TG thoracic ganglion, CG cerebral ganglion, JO Johnston’s organ, OM ommatidia, OC oocyte, OV ovaries.

Whole mosquitoes at 14 dpi (*n* = 3–5 per group) were analyzed by immunohistochemistry (IHC) using the anti-RVFV N antibody. Specific signals of N antigens could be detected under brightfield microscopy, while those signals could be better contrasted against hematoxylin background staining via Tetramethyl Rhodamine Iso-Thiocyanate (TRITC) fluorescent filter. RVFV N antigens were not detectable in mock-infected mosquitoes (Fig. 3a, b, e, and f), while they were abundantly detected in rMP-12-infected mosquitoes (Fig. 3c, d, g, and h). In rMP-12-infected mosquitoes, N antigens were detected in fat bodies throughout the body, while they were also found in cerebral ganglion and ommatidia (Fig. 3i), salivary gland (Fig. 3j), or anterior and posterior midgut epithelial cells (Fig. 3g, k). In rMP12-GM50-infected mosquitoes, *N* antigens were found in cerebral ganglion, ommatidia (Fig. 3l), fat bodies (Fig. 3m), or posterior midgut epithelial cells (Fig. 3n). In RVax-1-infected mosquitoes, N antigens were detectable in posterior midgut epithelial cells, but not in fat body or any other tissues outside midgut (Fig. 3o–q).

### Immunogenicity and protective efficacy of RVax-1 in inbred C57BL/6 mouse model

**Fig. 4:**
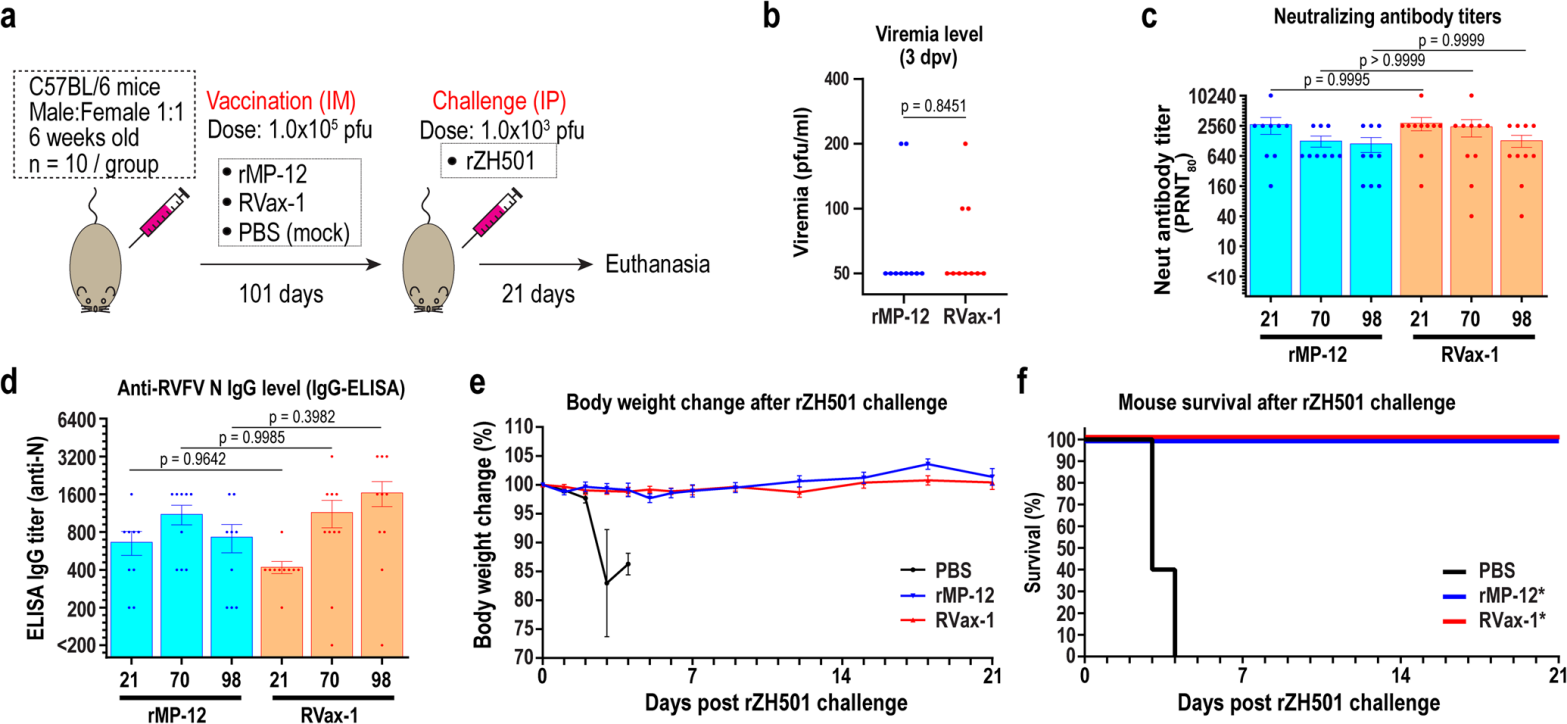
Immunogenicity and protective efficacy of rMP-12 and RVax-1 in C57BL/6 mice. **a** Schematic representation of vaccination and challenge experiment. C57BL/6 mice were IM mock-vaccinated or vaccinated with 1 × 10^5^ PFU of rMP-12 or RVax-1 (*n* = 10 per group), followed by rZH501 challenge at 101 dpv. One rMP-12-vaccinated animal died accidentally at 21 dpv during anesthesia. **b** Serum virus titers (PFU/ml) at 3 dpv are shown. Two vaccine groups were compared by unpaired *t*-test (*t* = 0.1982, df = 18). **c** Plaque Reduction Neutralizing Test 80 (PRNT_80_) neutralizing antibody titers at 21, 70, or 98 dpv are shown in a scattered dot plot graph with the means ± standard errors. Means were compared by one-way ANOVA followed by Tukey’s multiple comparison test (*F* = 1.149, df = 5). **d** ELISA IgG titers at 21, 70, and 98 dpv, and 21 dpc are shown in a scattered dot plot graph with the means ± standard errors. Means were compared by one-way ANOVA followed by Tukey’s multiple comparison test (*F* = 2.186, df = 5). **e** Body weight changes after rZH501 challenge (means ± standard errors) are shown. **f** The graph represents Kaplan–Meier survival curves after rZH501 challenge. Asterisks represent statistically significant differences (*p* < 0.01) based on log-rank testing compared to the mock-vaccinated group.

C57BL/6 mice were intramuscularly (IM) mock-vaccinated or vaccinated with 1 × 10^5^ PFU of rMP-12 or RVax-1, followed by a challenge with a lethal dose of the rZH501 strain at 101 days post vaccination (dpv) (Fig. 4a). Titers of rMP-12, RVax-1, or rZH501 inocula were 2.05 × 10^5^, 1.45 × 10^5^, or 2.16 × 10^2^ PFU, respectively, as determined by back-titration. At 3 dpv, two of ten mice vaccinated with rMP-12, and three of ten mice vaccinated with RVax-1, had detectable viremia (100–200 PFU/ml) (Fig. 4b). The mean Neutralizing antibody (PRNT_80_) titers were 2720 (21 dpv), 1280 (70 dpv), and 1120 (98 dpv) for rMP-12-vaccinated mice, while titers were 2896 (21 dpv), 2452 (70 dpv), and 1300 (98 dpv) for RVax-1-vaccinated mice (Fig. 4c). The mean IgG titers measured by IgG-ELISA using recombinant RVFV N proteins were 667 (21 dpv), 1111 (70 dpv), and 733 (98 dpv) for rMP-12-vaccinated mice, while titers measured 420 (21 dpv), 1150 (70 dpv), and 1650 (98 dpv) for RVax-1-vaccinated mice (Fig. 4d). There were no statistically significant differences in PRNT_80_ or anti-N IgG titers between RVax-1 and rMP-12 vaccination groups (one-way ANOVA followed by Tukey’s multiple comparison test). After rZH501 challenge, all mice vaccinated with rMP-12 or RVax-1 survived for 21 days without detectable clinical signs of diseases, while all mock-vaccinated mice succumbed to rZH501 infection by 4 days post challenge (dpc) (Fig. 4e, f). The mean IgG titers measured by IgG-ELISA using recombinant RVFV N proteins were 667 (21 dpv), 1111 (70 dpv), and 733 (98 dpv) for rMP-12-vaccinated mice, while titers measured 420 (21 dpv), 1150 (70 dpv), and 1650 (98 dpv) for RVax-1-vaccinated mice (Fig. 4d). There were no statistically significant differences in PRNT_80_ or anti-N IgG titers between RVax-1 and rMP-12 vaccination groups (one-way ANOVA followed by Tukey’s multiple comparison test).

**Fig. 5:**
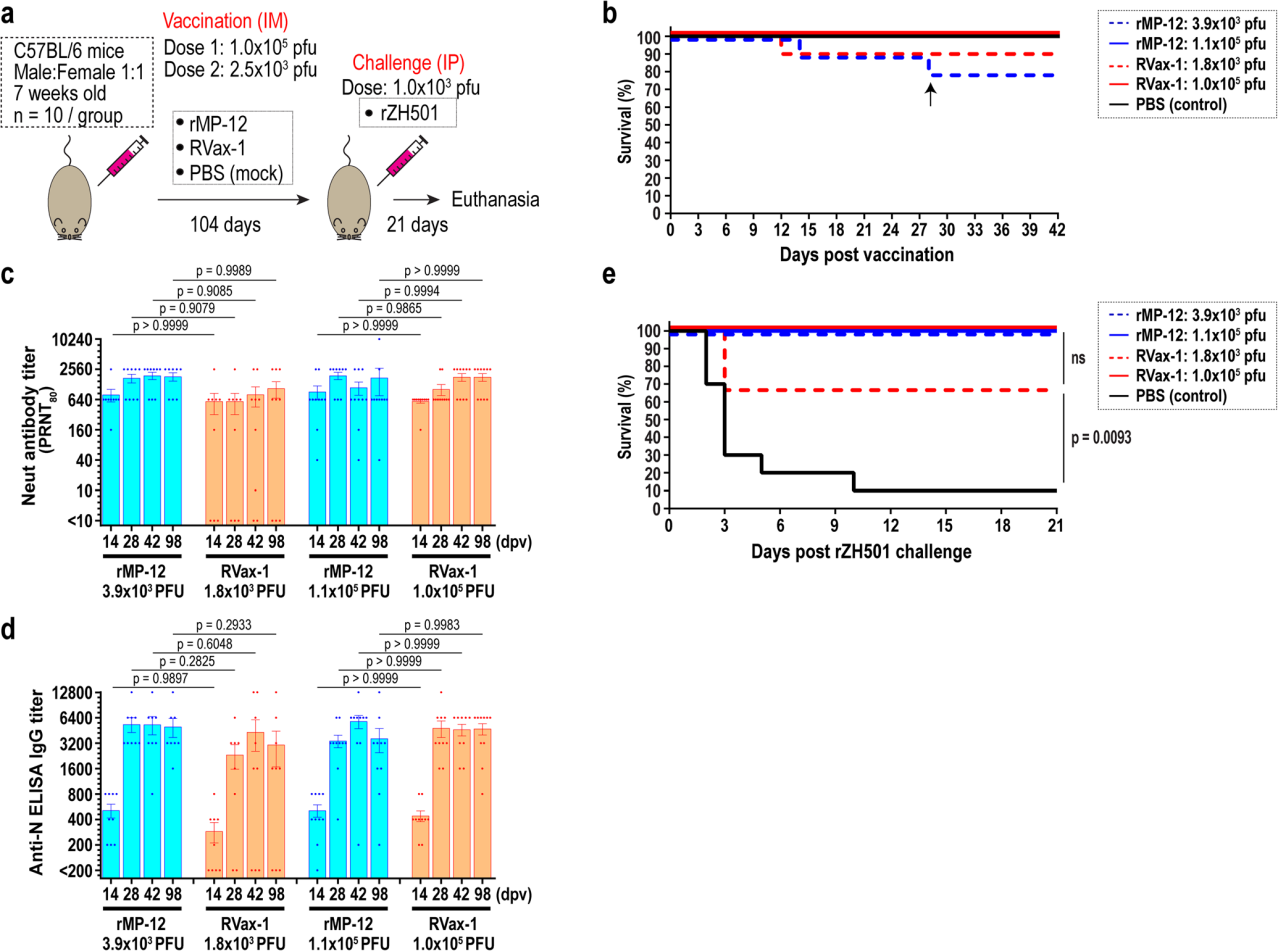
Immunogenicity and protective efficacy of low doses of rMP-12 or RVax-1 in C57BL/6 mice. **a** Schematic representation of vaccination and challenge experiment. C57BL/6 mice were IM mock-vaccinated or vaccinated with 2.5 × 10^3^ or 1.0 × 10^5^ PFU of rMP-12 or RVax-1 (*n* = 10 per group), followed by rZH501 challenge at 104 dpv. **b** The graph represents Kaplan–Meier survival curves after vaccination. Actual vaccine doses were determined by back-titration of inocula. An arrow at 28 dpv indicates an accidental death of animal during anesthesia, **c** Plaque Reduction Neutralizing Test 80 (PRNT_80_) neutralizing antibody titers (means ± standard errors) at 14, 28, 42, and 98 dpv are shown in a scattered dot plot graph with the means ± standard errors. Means were compared by one-way ANOVA followed by Tukey’s multiple comparison test (*F* = 1.663, df = 20). **d** IgG ELISA titers at 14, 28, 42, and 98 dpv are shown in a scattered dot plot graph with the means ± standard errors. Means were compared by one-way ANOVA followed by Tukey’s multiple comparison test (*F* = 2.367, df = 15). **e** The graph represents Kaplan–Meier survival curves after rZH501 challenge. Statistical comparisons based on log-rank testing are also shown (ns not significant).

To further evaluate the immunogenicity of RVax-1, we repeated the experiment by including 14, 28. 42, and 98 dpv samples. At this time, two doses (low dose: 2.5 × 10^3^ PFU; standard dose: 1.0 × 10^5^ PFU) of RVax-1 and rMP-12 were tested (Fig. 5a). Back titrations of inocula measured actual virus doses as 3.9 × 10^3^ and 1.0 × 10^5^ PFU for rMP-12, 1.8 × 10^3^ and 1.1 × 10^5^ PFU for RVax-1, and 4.6 × 10^3^ PFU for rZH501. At 12 and 14 dpv, mice vaccinated with 3.9 × 10^3^ PFU (rMP-12) or 1.8 × 10^3^ PFU (RVax-1) were found dead or humanely euthanized due to neurological clinical signs, respectively (Fig. 5b). An additional mouse (rMP-12-vaccinated, 3.9 × 10^3^ PFU) also died accidentally during anesthesia at 28 dpv. At the low dose, the mean PRNT_80_ titers were 800 (14 dpv), 1707 (28 dpv), 1920 (42 dpv), and 1840 (98 dpv) for rMP-12-vaccinated (3.9 × 10^3^ PFU) mice, while titers were 588 (14 dpv), 588 (28 dpv), 802 (42 dpv), and 1,068 (98 dpv) for RVax-1-vaccinated (1.1 × 10^5^ PFU) mice (Fig. 5c). Three non-responders were found for the low dose RVax-1-vaccinated mice. At the standard dose, the mean PRNT_80_ titers were 916 (14 dpv), 1920 (28 dpv), 1108 (42 dpv), and 1732 (98 dpv) for rMP-12-vaccinated (1.0 × 10^5^ PFU) mice, while titers were 592 (14 dpv), 1024 (28 dpv), 1792 (42 dpv), and 1792 (98 dpv) for RVax-1-vaccinated (1.1 × 10^5^ PFU) mice (Fig. 5c). The mean IgG titers measured by IgG-ELISA using recombinant RVFV N proteins were as follows: Low dose groups: 511 (14 dpv), 5333 (28 dpv), 5300 (42 dpv), and 5000 (98 dpv) for rMP-12-vaccinated group (3.9 × 10^3^ PFU) or 289 (14 dpv), 2325 (28 dpv), 4300 (42 dpv), and 3056 (98 dpv) for RVax-1-vaccinated group (1.8 × 10^3^ PFU); Standard dose group: 510 (14 dpv), 3400 (28 dpv), 5780 (42 dpv), and 3620 (98 dpv) for rMP-12-vaccinated group (1.0 × 10^5^ PFU) or 440 (14 dpv), 4800 (28 dpv), 4622 (42 dpv), and 4720 (98 dpv) for RVax-1-vaccinated group (1.1 × 10^5^ PFU) (Fig. 5d). Three non-responder mice without detectable neutralizing antibodies did not have ≥200 ELISA IgG titers. There were no statistically significant differences in PRNT_80_ or anti-N IgG titers between RVax-1 and rMP-12 vaccination groups. After the challenge with pathogenic rZH501 virus, 90% of mock-vaccinated mice and three RVax-1-vaccinated non-responder mice succumbed to infection, while all other mice survived the challenge (Fig. 5e).

### Viral load and pathophysiological changes of mock or vaccinated C57BL/6 mice at 3  days post rZH501 challenge

**Fig. 6:**
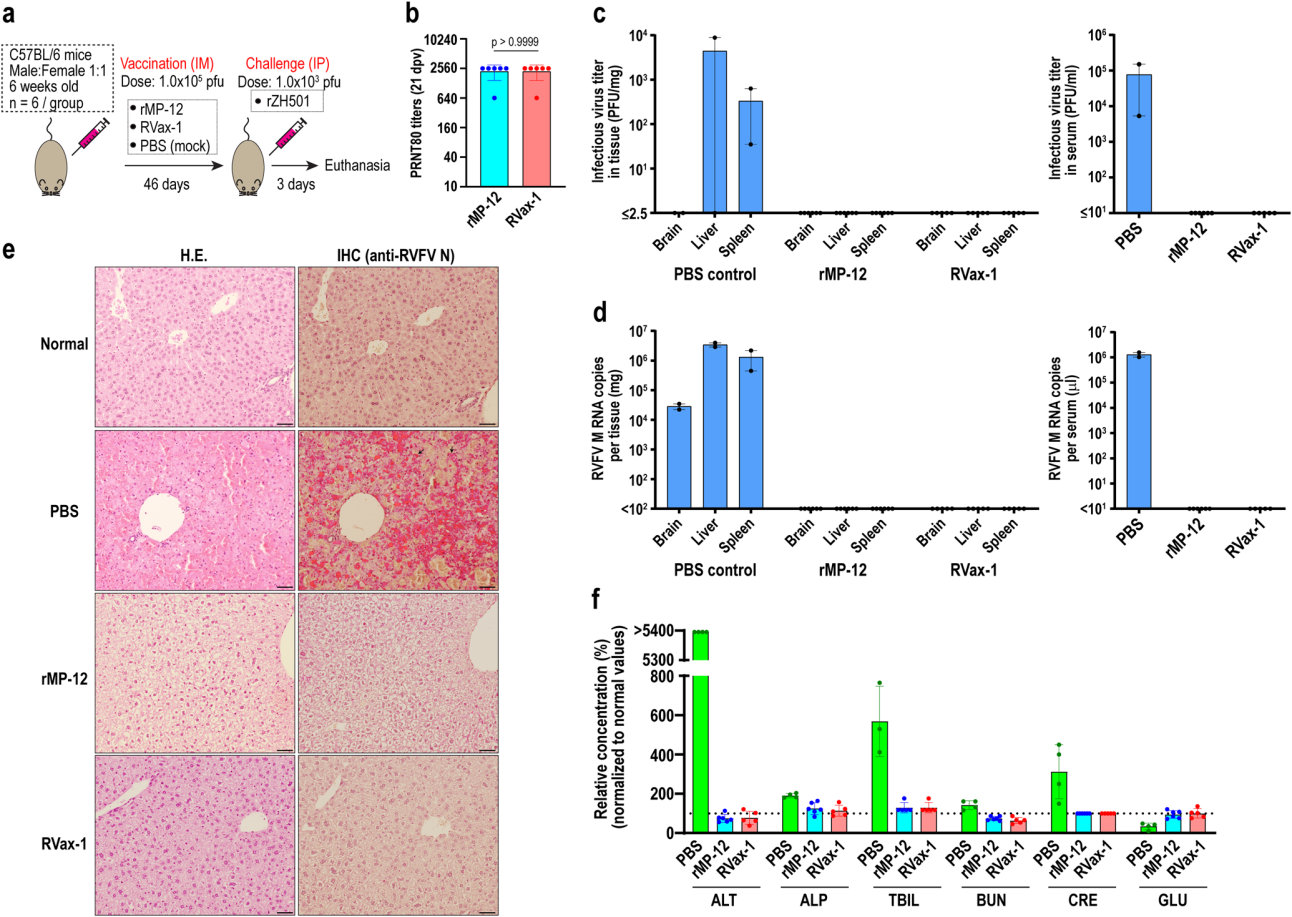
Viral load and pathophysiological changes of mock and vaccinated C57BL/6 mice at 3 days post rZH501 challenge. **a** Schematic representation of the vaccination and challenge experiment. C57BL/6 mice were IM mock-vaccinated or vaccinated with 1.0 × 10^5^ PFU of rMP-12 or RVax-1 (*n* = 6 per group), followed by rZH501 challenge at 46 dpv. All surviving mice were humanely euthanized at 3 days post rZH501 challenge (dpc). All mock-vaccinated mice died at 2 dpc, among which only two animals were available for virus titers, qRT-PCR, and histopathology examinations. One RVax-1-vaccinated animal died accidentally at 42 dpv during anesthesia. **b** Plaque Reduction Neutralizing Test 80 (PRNT_80_) antibody titers at 21 dpv are shown in a scattered dot plot graph with means ± standard deviations. Means were compared by unpaired *t*-test (*t* = 0, df = 10). **c** Infectious virus titers in brains, livers, and spleens (left panel), or sera (right panel) by plaque assay. Scattered dot plot graphs with means ± standard errors of tested samples are shown. **d** Copy number of viral M segment RNA was measured by Taqman qRT-PCR using the standard curve for in vitro synthesized RVFV M segment RNA. Scattered dot plot graphs with means ± standard errors of tested samples are shown. **e** H.E. stain or immunohistochemistry using anti-RVFV N antibody (IHC) for liver sections from untreated normal C57BL/6 mice, or rZH501-challenged, mock-vaccinated (PBS) or vaccinated (rMP-12 or RVax-1) mice. Arrows represent N antigen-positive cells in IHC. Bars represent 50 µm. **f** Relative concentration (% to the normal reference values) of alanine aminotransferase (ALT), albumin, alkaline phosphatase (ALP), total bilirubin (TBIL), blood urea nitrogen (BUN), creatinine (CRE), and glucose (GLU) in mouse blood at 3 dpc are shown in a scatter dot plot graph with the means ± standard deviations. Blood samples from four C57BL/6 mice that died at 3 days post rZH501 challenge in another experiment (Fig. 5) were used as the mock-vaccinated (PBS) control for the comparison.

To characterize early pathological changes and virus replication in mock-vaccinated or vaccinated groups upon rZH501 challenge, C57BL/6 mice were IM mock-vaccinated or vaccinated with 1 × 10^5^ PFU of rMP-12 or RVax-1, followed by a challenge with a lethal dose of rZH501 at 46 dpv (Fig. 6a). Titers of rMP-12, RVax-1, or rZH501 inocula were 4.5 × 10^5^, 2.8 × 10^5^, or 1.6 × 10^3^ PFU, respectively, as determined by back-titration. Mean PRNT_80_ neutralizing antibody titers at 21 dpv were 2240 for both rMP-12 and RVax-1 groups (Fig. 6b). After rZH501 challenge, four mock-vaccinated mice succumbed to infection early at 2 dpc, whereas two remaining mice were euthanized due to observed sickness at the follow-up observation at 2 dpc. We included the latter two mice in the follow-up analysis of this group. In tissue samples collected from mock-infected and rZH501-challenged mice at 2 dpc, viruses were detected in sera (7.8 × 10^4^ PFU/ml, 1.3 × 10^6^ RNA copies/µl), liver (8.8 × 10^3^ PFU/mg tissue, 3.4 × 10^6^ copies/mg tissue), spleen (3.3 × 10^2^ PFU/mg tissue, 1.3 × 10^6^ copies/mg tissue), and brain (<2.5 PFU/mg tissue, 2.8 × 10^4^ copies/mg tissue) (Fig. 6c, d). Contrastingly, viruses were not detectable at 3 dpc from sera, brain, liver, or spleen samples of mice vaccinated with rMP-12 or RVax-1 (detection limit of infectious RVFV was 2.5 PFU/mg in tissue and 10 PFU/ml in sera, and the detection limit of RVFV RNA was 100 copies/mg tissue and 10 copies/µl sera) (Fig. 6c, d). Histopathological examination of liver sections of mock-vaccinated mice showed diffuse necrosis of hepatocytes, whereas vaccinated mouse livers showed mild ballooning regeneration of hepatocytes without any detectable necrotic changes or inflammatory cell infiltrations (Fig. 6e, left panels). Immunohistochemistry using anti-RVFV N antibody detected abundant viral antigens in residual hepatocytes in mock-vaccinated RVFV-challenged mice, but not in those vaccinated with rMP-12 or RVax-1 (Fig. 6e, right panels). Viral antigens were also detected in mononuclear cells in spleens but not in brains of mock-vaccinated animals, whereas no viral antigens were found in those vaccinated with rMP-12 or RVax-1 (Supplementary Figure 3). Blood samples were further tested for clinical markers to identify organ damage. Since whole blood samples were not available for the mock-vaccinated group, we alternatively tested four blood samples of mock-vaccinated and rZH501-challenged C57BL/6 mice collected at 3 dpc in a repeated mouse experiment (Fig. 5). Mock-vaccinated mice showed high levels of alanine aminotransferase (ALT) (beyond measurable range, >5400% vs. normal), alkaline phosphatase (ALP) (mean 190% vs. normal), total bilirubin (mean 569% vs. normal), creatinine (mean 313% vs. normal), and blood urea nitrogen (143% vs. normal), whereas the glucose level was reduced (mean 34% vs. normal) (Fig. 6f). Mice vaccinated with either rMP-12 or RVax-1 showed normal values for those markers at 3 dpc.

## Discussion

The RVax-1 candidate vaccine encodes all mutations of the parental MP-12 strain, while additionally encoding 36, 37, 167, and 326 mutations in N, NSs, M, and L ORFs, respectively^24^, and an in-frame deletion of the NSm and 78kD genes in the M segment^10,26^. The attenuation of the original MP-12 strain is supported by a few amino acid changes in M and L segments^16,18^. Therefore, a reassortment between the MP-12 and pathogenic wild-type RVFV strains might create new pathogenic genotypes of RVFV through MP-12 S and/or L segments. Silent mutations derived from rMP12-GM50 contribute to attenuation of reassortant RVFV via its S, M, or L segment^24^. Additionally, a unique silent mutation pattern at every 50 nt supports follow-up evaluation of viral reassortment or recombination via RVax-1 in nature if it occurs. This study demonstrated (i) the genetic stability of 566 silent mutations of RVax-1 during viral passages in Vero cells, (ii) a single dose vaccine potency of RVax-1, which can confer full protection of mice from lethal pathogenic RVFV challenge, and (iii) inefficient systemic dissemination of RVax-1 in mosquito vectors.

Neutralizing antibodies play a major role in protection from lethal RVF diseases^27–29^, while cellular immune responses also contribute^30^. Passive transfer of immune sera with a final PRNT_80_ titer of 1:10 to 1:20 can protect animals from lethal RVFV disease^28^. Thus, neutralizing antibody titers can be used as a surrogate endpoint of the immunological protection in RVFV infections. In this study, we analyzed the neutralizing antibody titers induced by RVax-1 or parental rMP-12 over 100 day-period, while a lethal challenge using rZH501 was performed at the end of study. In the Phase 2 trial of the MP-12 vaccine, a single dose IM vaccination with 1 × 10^5^ PFU of MP-12 led to the peak PRNT_80_ titers between 1:60 and 1:1920 with one non-responder at 28 dpv (*n* = 18), while PRNT_80_ titer of 1:20 was maintained for 5 years in 89% of participants (*n* = 9)^14^. In our study, C57BL/6 mice IM vaccinated with 1 × 10^5^ PFU of RVax-1 induced PRNT_80_ titers of 1:2896, 1:2452, and 1:1300 at 21, 70, and 98 dpv, respectively, which were comparable to those induced by rMP-12 vaccination, which were 1:2720, 1:1280, and 1:1,120 at 21, 70, and 98 dpv, respectively. The immunogenicity and protective efficacy of RVax-1 was thus indistinguishable from rMP-12 at 1 × 10^5^ PFU dose. We also tested whether a reduced dose of RVax-1 would abrogate the protective immunity or not. Past study showed that vaccinations with 3 × 10^3^ PFU or 1.35 × 10^6^ PFU of MP-12 could induce mean PRNT_70_ titers of 1:25 or 1:278 in sheep, respectively^20^. Our vaccination experiment using 1.8 × 10^3^ PFU of RVax-1 resulted in PRNT_80_ titers of 1:588, 1:588, 1:802, and 1:1,068 at 14, 28, 42, and 98 dpv, respectively, while the application of 3.9 × 10^3^ PFU of rMP-12 led to 1:800, 1:1707, 1:1920, and 1:1840 at 14, 28, 42, and 98 dpv, respectively. Three non-responders were found in mice vaccinated with RVax-1, but not with rMP-12, all of which succumbed to infection after rZH501 challenge. These results confirmed that the presence of neutralizing antibodies is a predictable clinical marker for the protection from RVFV^13,14^. The immunogenic effect was similar for both RVax-1 and rMP-12 vaccinations, while the relevance of vaccine non-responders to the RVax-1 doses should be further evaluated in future studies.

Although vaccinated animals did not show any clinical signs of disease after pathogenic RVFV challenge, pathological changes or viral loads after the challenge have not been studied. Therefore, mice vaccinated with rMP-12 or RVax-1 were challenged at 46 dpv with pathogenic rZH501, followed by tissue sampling at 3 dpc. Unexpectedly, all mock-vaccinated animals died at 2 dpc in the experiment. Mock-vaccinated mice showed histopathological lesions in livers and spleens accompanied by abundant viral antigens and succumbed to infection at 2 dpc. Mice vaccinated with RVax-1 or rMP-12 did not show remarkable histological changes other than mild ballooning regeneration of hepatocytes after rZH501 challenge. In IHC, viral antigens were not detectable in livers, spleens, and brains in those vaccinated and challenged mice. Furthermore, none of those vaccinated mice harbored infectious virus or viral RNA in sera, spleen, or brain at 3 dpc. Thus, those results showed that mice vaccinated with rMP-12 or RVax-1 could prevent histopathological changes and early replication of pathogenic RVFV in bloods, livers, or spleens.

Mice are highly susceptible to RVFV infection, in which the LD_50_ for rZH501 strain was reported as 5.6 PFU^12,31^. Although MP-12 strain is highly attenuated in mice (LD_50_ ≥ 2.0 × 10^6^ PFU)^12^, sporadic incidence of viral neuroinvasion and the resulting CNS disease can occur in vaccinated adult mice^32^. Although there is a limitation in terms of the translation of finding into humans, mouse experiments might be useful to compare relative neuroinvasiveness (e.g., occurrence of viral CNS disease, viral spread into CNS) and neurovirulence (e.g., histological lesions in CNS, mean survival time) between vaccine strains at a low cost. As reported in previous study^14^, 19-day-old mice will be useful for further evaluation of RVax-1 and parental rMP-12 strain in this regard due to their age-dependent susceptibility. Nevertheless, neurovirulence of RVax-1 candidate vaccine should be further validated in nonhuman primate model as described for parental MP-12 strain because of their susceptibility to RVFV like that in humans^33^.

The primary vector for RVFV is considered floodwater *Aedes* spp. (e.g., *Ae. mcintoshi*, *Ae. vexans*), which can vertically transmit RVFV in Africa. RVFV was isolated from both female and male *Ae. mcintoshi* mosquitoes hatched from eggs collected in Kenya^34^. RVFV has also been isolated from more than 53 mosquito species, including *Ae. aegypti*^35^. *Ae. aegypti* has been considered a moderately competent vector, with an infectious dose of 10^≥8.0^ PFU/ml for efficient dissemination of RVFV (75% dissemination rate at day 11–16)^9,11,36,37^. In contrast, early dissemination of RVFV occurred in *Cx. pipiens* and *Ae. mcintoshi* at 3–10 dpi, yet the dissemination rates were less than 50% until 18 dpi even after 10^≥8.0^ PFU/ml input^36^. Therefore, *Ae. aegypti* can serve as an excellent model to study the dissemination of RVFV. We evaluated three pools of 10 mosquitoes per group to measure viral RNA loads in abdomens, legs, and heads/thoraxes/wings. The RVax-1 M segment RNA copies were 76.2-, 67.9-, or 759.0-fold less than those of rMP-12 in abdomen, leg, or head/thorax/wing, respectively, at 14 dpi. Further immunohistochemical characterization of those mosquitoes showed that viral antigens of rMP-12 or rMP12-GM50 distribute not only midgut epithelial cells but also fat bodies, cerebral ganglion, ommatidia, or salivary gland, whereas RVax-1 was detectable only in midgut epithelial cells. Our study showed that RVax-1 does not disseminate efficiently in *Ae. aegypti* mosquitoes via oral infection, confirming that 78kD/ NSm protein play an important role in viral dissemination in mosquitoes as reported by others previously^9,11,37^. Although it is considered that a low level of viremia upon the vaccination with MP-12 is not sufficient to transmit MP-12 virus into mosquitoes^20^, the use of RVax-1 candidate vaccine will further minimize potential risk of viral transmission via natural mosquito vectors.

The characteristic pattern of RVax-1 silent mutations, which are not found in natural RVFV strains, will be useful for tracing potential environmental spill-over of the RVax-1 strain upon field use (e.g., in natural reassortant or recombinant strains). Serial viral passages of RVax-1 in Vero cells did not lead to detectable reversion changes at MP-12-specific mutation sites (*n* = 23), or nucleotide changes at RVax-1-specific silent mutation sites (*n* = 566). We could detect minor changes (≤0.6% in viral subpopulations) for 25 of 566 silent mutations, while it would not affect overall vaccine quality if we will use a seed lot system. Further studies will be required for the genetic stability of those silent mutation markers in reassortant strains derived from RVax-1 and wild-type RVFV strains in mosquitoes. Overall, these findings demonstrated the Proof of Concept for RVax-1 as a next-generation MP-12 vaccine, which will support further preclinical and clinical developments.

## Methods

### Media, cells, and viruses

Vero cells (*Chlorocebus* sp., kidney epithelial cells, ATCC CCL-81) or MRC-5 cells (human lung diploid cells, ATCC CCL-171) were maintained in DMEM (Gibco, Thermo Fisher Scientific Inc., Waltham MA), containing 10% fetal bovine serum (FBS, HyClone, GE Healthcare, Chicago IL), penicillin (100 U/ml, Gibco), and streptomycin (100 µg/ml, Gibco), in a humidified cell culture incubator (5% CO_2_, 37 °C). BHK-21 cells (*Mesocricetus auratus*, baby hamster kidney fibroblast cells, ATCC CCL-10) were maintained in minimum essential medium (MEM) alpha containing 10% FBS, penicillin (100 U/ml), and streptomycin (100 µg/ml) at 37 °C with 5% CO_2_, while BHK cells that stably express T7 RNA polymerase (BHK/T7-9 cells)^38^ were maintained with hygromycin B (600 μg/ml). C6/36 cells (*Ae. albopictus*, ATCC CRL-1660) were maintained at 28 °C without CO_2_ in Leibovitz’s L-15 medium containing 10% FBS, 10% tryptose phosphate broth (TPB), penicillin (100 U/ml), and streptomycin (100 µg/ml). Cells used in this study were verified to be mycoplasma free at the University of Texas Medical Branch at Galveston (UTMB) Tissue Culture Core Facility, and the identities of MRC-5 cells were authenticated by Short Tandem Repeat analysis (UTMB Molecular Genomics Core Facility).

The MP-12 vaccine Lot 7-2-88 was amplified once in MRC-5 cells for use in this study. RVax-1 or rMP-12 viruses were recovered from Vero cells via reverse genetics^25^. For mosquito study, the rMP-12, RVax-1, or rMP12-GM50 viruses^24^ were further amplified at 37 °C in BHK-21 cells, followed by concentration using the Centricon Plus-70 (Sigma-Aldrich). The arMP12-∆NSm21/384 strain (deletion of the M-segment sequence at nt. 21 – 384) was described previously^26^. The titration of RVFV was performed by a plaque assay using Vero cells with crystal violet staining^25^.

### Plasmids

The pProK-sPI-vS(+), pProK-sPI-vM(+), and pProK-sPI-vL(+), which encode the full-length antiviral-sense S, M, and L-segments of the RVFV MP-12 strain, respectively, immediately downstream of the *Macaca mulatta* precursor rRNA gene (RNA polymerase I) promoter, were described previously^25^. These plasmids were further modified to encode the full-length antiviral-sense S, M, and L-segments of RVax-1 virus, respectively, creating plasmids pProK-sPI-vS(+)-RVax, pProK-sPI-vM(+)-RVax, and pProK-sPI-vL(+)-RVax. Helper plasmids expressing MP-12 N, L, or GnGc proteins (pCAGGSK-vN, pCAGGSK-vL, and pCAGGSK-vG, respectively) were described previously^25^.

### Rescue of recombinant RVax-1 infectious clones

For the rescue of RVax-1 clone, Vero cells in 6-well plates (1 × 10^6^ cells/well) were transfected with 0.8 µg of pProK-sPI-vS(+)-RVax, 0.8 µg of pProK-sPI-vM(+)-RVax, 0.8 µg of pProK-sPI-vL(+)-RVax, 0.6 µg of pCAGGSK-vN, 0.5 µg of pCAGGSK-vL, and 0.5 µg of pCAGGSK-vG, using 12 µl of the TransIT-293 Transfection reagent. Culture media was replaced at 24 hpt, followed by the transfer of cell and media into a 10-cm culture dish at 72 hpt for further incubation at 37 °C. Culture supernatants were collected when transfected cells showed cytopathic effects.

### Stability of RVax-1 silent mutations during serial passages

Vero cells were infected with P0 stock of RVax-1 at a multiplicity of infection (MOI) of 0.01 in 12-well plates, in triplicate. Culture supernatants were collected at 3 dpi, and 3 µl of supernatants were transferred into fresh Vero cells. Upon completion of 10 serial passages, virus titers of culture supernatants at each passage were determined. Vero cells were then infected with P4 and P9 supernatants at MOI 0.01, and total RNA samples were extracted at 48 hpi using RNeasy (QIAGEN), which were designated as P5 and P10 samples, respectively. The RNA-Seq library of whole transcripts was constructed from total RNA (1.0 µg). Next-generation sequencing of total RNA (75-bp paired-end library sequencing) was performed using the NextSeq 550 sequencing platform (Illumina, Inc., San Diego, CA) at the Next Generation Sequencing Core Facility at UTMB. The FASTAQ reads were aligned with L, M, or S segment sequences for the RVFV rMP12-GM50 strain (GenBank #MF593928.1, MF593929.1, MF593930.1), with the similarity fraction = 0.8, mismatch cost = 2, insertion cost = 3, and deletion cost = 3. Subsequently, genetic variants (≥0.1%) were screened using the Basic Variant Detection tool in the CLC Genomics Workbench program, with the neighborhood radius = 5, minimum central quality = 25, minimum neighborhood quality = 15, and active read direction filter. Then, variants (≥0.1%) that met the criteria of a forward/reverse balance of 0.25–0.75, ≥10 independent counts, and ≥ 20 average quality, were listed. From these, RVax-1-specific 566 silent mutation sites were evaluated for genetic subpopulation changes at P5 and P10 in Vero cells.

### Analysis of viral dissemination in infected *Aedes aegypti*

Four-hundred female *Ae. aegypti* Orlando mosquitoes (3–5-day old) were fed on a combination of defibrinated sheep blood spiked with concentrated virus (rMP-12, rMP12-GM50, or RVax-1), or with DMEM (mock-infected control), at a 2:1 ratio. Blood feeding was performed using commercial sausage casing stretched over Hemotek feeding reservoirs attached to feeders which were calibrated to 37 °C. Remaining blood was collected from the feeders to perform back titrations. Mosquitoes were allowed to feed for 1 h, after which, mosquitoes were cold anesthetized and separated based on feeding status. Engorged females were placed back into cartons and fed on a 10% sucrose solution for the duration of the extrinsic incubation period. At 7 and 14 dpi, dead mosquitoes were removed from each carton. Abdomen, leg, or head/thorax/wing from three pools (*n* = 10 per pool) of mosquitoes in each group were separately collected into 2 ml tubes filled with Trizol. Tissues were homogenized with 2.8 mm stainless steel beads (Millipore-Sigma), followed by total RNA extraction using Direct-zol RNA Miniprep Kit (Zymo Research) for qRT-PCR analysis of viral RNA loads. Whole mosquitoes (*n* = 3 to 5 per group) were fixed in 10% neutral buffered formalin for immunohistochemical analysis of RVFV N antigens.

### Immunogenicity and protective efficacy of RVax-1 in C57BL/6 mice

A mouse experiment was performed to evaluate (i) viremia at 3 dpv, (ii) neutralizing antibody and ELISA IgG levels after vaccination, and (iii) protective efficacy against a lethal dose challenge of rZH501, in mock-vaccinated and vaccinated mice. Six-week-old C57BL/6 mice (Charles River) were vaccinated IM with PBS (mock), or 1 × 10^5^ PFU of rMP-12 or RVax-1 (five males and five females per group: not randomized). Mice were subsequently challenged with 1 × 10^3^ PFU of pathogenic rZH501 strain intraperitoneally (IP) at 101 dpv. Mice were observed daily for 21 days after challenge, and individual body weights were measured daily for seven days, and then every three days thereafter. Mice showing more than 20% body weight loss, and/or showing clinical signs such as viral encephalitis or severe lethargy, were humanely euthanized, while all surviving mice were humanely euthanized at 21 dpc. Sera were collected at 3, 21, 70, and 98 dpv via the retro-orbital vein, and at 21 dpc via cardiac puncture. This experiment was repeated once with two different doses of rMP-12 or RVax-1 (2.5 × 10^3^ PFU or 1 × 10^5^ PFU: five males and five females per group: not randomized). Mice were IP challenged with 1 × 10^3^ PFU of pathogenic rZH501 strain at 104 dpv. Mice were observed daily for 21 days after challenge. Sera were collected at 14, 28, 42, and 98 dpv via the retro-orbital vein, and at 21 dpc via cardiac puncture.

Pathological changes of vaccinated C57BL/6 mice upon rZH501 challenge. A mouse experiment was performed to evaluate (i) viral load at 3 dpc, and (ii) liver injury at 3 dpc in mock-vaccinated and vaccinated mice. Six-week-old C57BL/6 mice (Charles River, three males and three females per group: not randomized) were IM vaccinated with PBS (mock), rMP-12 (1.0 × 10^5^ PFU) or RVax-1 (1.0 × 10^5^ PFU). Mice were subsequently challenged with 1 × 10^3^ PFU of pathogenic rZH501 strain (IP) at 46 dpv. Mice were observed daily for 3 days after challenge, and all surviving animals were humanely euthanized at 3 dpc. Mice showing more than 20% body weight loss, and/or showing clinical signs such as viral encephalitis or severe lethargy, were humanely euthanized. Sera were collected at 3, 21, and 42 dpv via the retro-orbital vein, and at 3 dpc via cardiac puncture.

### Measurement of viral M RNA copies by qRT-PCR

Total RNA from mouse or mosquito tissues were extracted using Direct-zol RNA Miniprep Kit (Zymo Research), while the concentration of extracted RNA was measured by the Qubit 2.0 Fluorometer (Thermo Fisher Scientific). Mouse sera mixed with Trizol were combined with 1.2 µg of in vitro transcribed chloramphenicol acetyltransferase RNA^17^ to evaluate the RNA copies per serum volume. The 1^st^ stranded cDNA were synthesized from 50 to 500 ng total RNA by iScript Reverse Transcriptase (BioRad), followed by PCR reaction with SsoAdvanced Universal Probes Supermix (BioRad) using the Mic qPCR Cycler (4 channel): initial denaturation at 98 °C for 5 min, 40 cycles of 98 °C for 15 s, 60 °C for 45 s, and final denaturation at 98 °C for 10 min. The PCR reaction targeted the 5' untranslated region (UTR) of RVFV M-segment by the forward primer (RV-MUTR-F: 5'- GCT TGT GAA TAT TCT AGT TGG CG -3'), the reverse primer (5'- CCG GTG CAA CTT CAA AGA GT -3'), and Taqman probe (5'FAM- ATC GTC TTT TGC CAG ATT AGC TG-3'BHQ1). This set of primers and probe was used for the detection of RVFV rMP-12, rMP12-GM50, RVax-1, or rZH501.

Serially 10-fold diluted M-segment RNA, generated by in vitro transcription from pProT7-vM(+) plasmid, were used for the 1st stranded RNA synthesis with iScript reverse transcriptase. Actual concentrations of RNA (copy number/µl in reaction) in each RNA dilution were measured by droplet digital PCR with the above-mentioned primers and probe set for RVFV M-segment 5' UTR using the QX100 droplet generator and reader according to the manufacturer’s instruction. The resulting cDNA set derived from serial RNA dilutions with known RNA concentrations were used for the validation of standard curve using the Mic qPCR.

### Histological examinations of RVFV-infected samples

Mouse livers, spleens or brains, or whole mosquitoes, were fixed with 10% neutral buffered formalin and embedded in paraffin blocks. Thin sections were then stained with hematoxylin-eosin staining (H.E.) at the Anatomic Pathology Laboratory at UTMB. For IHC, sectioned tissues were treated with proteinase K antigen retrieval solution (Abcam, Waltham MA), followed by blocking with Animal-Free Blocker (Vector Laboratories), anti-RVFV N rabbit polyclonal antibody^26^, and biotinylated secondary goat anti-rabbit IgG antibody (Vector Laboratories). Sections were further incubated with streptavidin alkaline phosphatase (Vector Laboratories) and then the ImmPACT Vector Red substrate (Vector Laboratories), before being additionally stained with hematoxylin to visualize the background. Images were captured via cellSens software using a DP74 camera attached to an Olympus IX73 microscope. Positive IHC signals were analyzed under brightfield (magenta) or TRITC fluorescent filter (red) according to manufacturer’s instruction. Merged images of brightfield (reduced brightness) and TRITC fluorescent filter were made by cellSens software.

### Plaque reduction neutralization test

For the plaque reduction neutralization test (PRNT_80_), each serially diluted four-fold mouse serum was transferred into 96-well plates containing approximately 50 PFU of MP-12 virus, followed by an incubation at 37 °C for 1 hour. Virus titers of this mixtures were measured by plaque assay using Vero cells. The average number of plaques visualized by crystal violet staining from at least five different wells from mock-immunized mice sera was used to set the cut-off number of 80% reduction.

### IgG-enzyme-linked immunosorbent assay (ELISA)

IgG ELISA was used for detection of anti-RVFV N antibody using His-tagged RVFV N proteins purified from *Escherichia coli*. The RVFV N ORF was cloned between restriction sites Sal-I and Not-I in the pET43.1b (+) plasmid (Millipore Sigma) which encodes N- and C-terminal His-tags. The sequence between restriction sites Spe-I and Sac-I, including the N-terminal His-tag, was removed from the plasmid, resulting in the plasmid designated pET43.1∆His-RVFV-N, which encodes the RVFV N protein tagged with an N-terminal Nus-tag and C-terminal His-tag. BL21 (DE3) competent *E. coli* cells, which were transformed with pET43.1∆His-RVFV-N, were cultured in LB medium at 37 °C for 16 h., and then induced by 100 µM isopropyl β-D-1-thiogalactopyranoside for 4 h. The RVFV N protein was incubated with Ni-NTA His-Bind Resin (Millipore Sigma) in the presence of 6 M urea, followed by washing and elution with 2 M urea. Purified RVFV N proteins were used for ELISA after the replacement of buffer with PBS by PD-10 desalting columns (GE Healthcare) and the concentration by Amicon Ultra15 centrifugation filter units (10 kDa cut-off, Millipore Sigma). ELISA plates were coated with 80 ng per well of RVFV N proteins overnight. Plate was washed three times with PBS containing 0.1% tween 20 (PBS-T), followed by bocking with PBS-T containing 0.5% BSA at 37 °C for 2 h. Wells were incubated with serially diluted serum samples at 37 °C for 1 h. After washing three times with PBS-T, wells were incubated with HRP-conjugated anti-mouse IgG (Santa Cruz Biotech) at 37 °C for 1 hr, followed by washing with PBS-T four times. At 30 min after adding ABTS solution (Sigma-Aldrich), the optical density (OD) at 405 nm was measured by Accuris SmartReader 96 (Accuris Instruments). Cut-off value of IgG ELISA was determined by geometric mean + 2x standard deviations of OD values of 12 normal sera per ELISA plate.

### Blood chemistry

VetScan Comprehensive Diagnostic Profile (Abaxis) was used for the measurement of murine ALT, ALP, creatinine, glucose, total bilirubin, and blood urea nitrogen in mouse blood at 3 dpc, according to the manufacturer’s instruction.

### Statistical analysis

Sample sizes were designed by G*Power 3.1.9.7. (Research Resource Identifiers: RRID:SCR_013726)^39^ so that the described group and measurement values will provide at least 80% power (*α* = 0.05) to detect a large effect size (*d* = 0.80). No data were excluded from analysis except for the lack of samples due to unexpected animal death. Measurements were taken using distinct biological samples. For comparisons among groups, differences were analyzed by two-tailed unpaired *t*-test (two groups), one-way ANOVA (>2 groups) followed by Tukey’s multiple comparison test, or two-way ANOVA (>2 groups). Same for comparisons among groups of viral titers or RNA copy numbers, arithmetic means of log_10_ values were analyzed by ANOVA. Survival curves of mice were analyzed by Kaplan-Meyer method. Relative diameter lengths from 20 randomly selected plaques of rMP-12 or RVax-1 were measured by Image J software 1.53a (RRID:SCR_003070) with the Cell Counter Plugin^40,41^, followed by statistical analysis of mean diameters by unpaired *t*-test. All statistical analyses were performed with GraphPad Prism 8.4.3. (RRID:SCR_002798).

### Ethics statement

All experiments using recombinant DNA and infectious RVFV have been performed upon the approval of the Notification of Use (#2021017 and #2019025) by the Institutional Biosafety Committee at UTMB. Mouse studies were performed in the UTMB Robert E. Shope or GNL BSL-4 laboratory accredited by the Association for Assessment and Accreditation of Laboratory Animal Care (AAALAC) in accordance with the Animal Welfare Act, NIH guidelines, and US federal law. Animal protocol #1912097 was approved by UTMB Institutional Animal Care and Use Committee (IACUC). All work with pathogenic rZH501 was performed in the Robert E. Shope or GNL BSL-4 laboratory, UTMB. All animal experiments were performed in unblinded manner for the safe handling of infectious samples. IBC approval #458 was obtained for mosquito experiments using RVFV MP-12 or the variant strains performed at SUNY Upstate Medical University. Domestic transportation of RVFV MP-12 or the variant strains between UTMB and SUNY Upstate Medical University was performed with the U.S. veterinary permit for importation and transportation of controlled materials and organisms and vectors approved by USDA.

### Availability of materials

Any unique biological materials used in this study can be available to others through the agreement term made via the Office of Technology Transfer at UTMB.

### Reporting summary

Further information on research design is available in the Nature Research Reporting Summary linked to this article.

## Supplementary information

Supporting Figures

REPORTING SUMMARY

## Data Availability

The full genome sequence of RVax-1 vaccine candidate can be found in GenBank (accession numbers ON211620 – ON211622). The raw RNA-Seq data are available via the Sequence Read Archive (SRA) database (the BioProject accession number: PRJNA825509).

## Materials availability

Any unique biological materials used in this study can be available to others through the agreement term made via the Office of Technology Transfer at UTMB.
