# Supplementary material for: Evaluations of rationally designed rift valley fever vaccine candidate RVax-1 in mosquito and rodent models
Source: NPJ Vaccines. 2022 Sep 21;7:109. doi: 10.1038/s41541-022-00536-3 (PMC9492667; doi:10.1038/s41541-022-00536-3)
Supplement: Supplementary file 1 — Supporting Figures [file 41541_2022_536_MOESM1_ESM.pdf]

Supplementary Figure 1

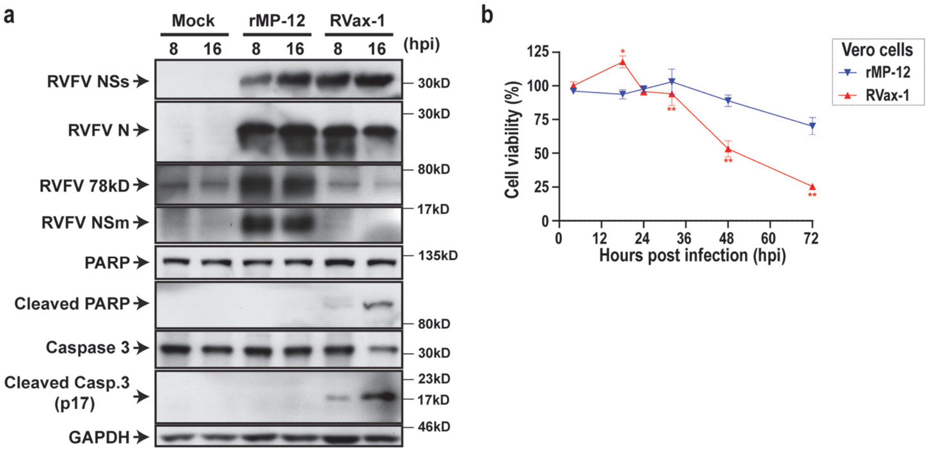

Supplementary Figure 1. Cell viability of Vero cells upon the infection with rMP-12 or RVax-1. Vero cells were mock-infected or infected with rMP-12 or RVax-1 at MOI 5. Cells were incubated at 37°C for the indicated period. **(a)** Western blots using anti-RVFV N rabbit polyclonal antibody, anti-RVFV NSs rabbit polyclonal antibody, anti-RVFV NSm/78kD rabbit polyclonal antibody raised against synthesized peptide, NH_2- CHGKDPEDKISLIKGPPHKKR-OH (custom antibody: RVF_NSm_53/73, ProSci Inc.), or antibodies against intact or cleaved caspase 3 (Cell Signaling Technology), intact or cleaved poly(ADP-ribose) polymerase (PARP) (Cell Signaling Technology), or GAPDH antibody (Thermo Fisher Scientific) (loading control). All blots were from the same experiment and processed in parallel. **(b)** Vero cell viability as measured by MTT (3-[4,5-dimethylthiazol-2-yl]-2,5-diphenyl tetrazolium bromide) assay (Millipore Sigma) according to manufacturer's instruction. At 1, 14, 20, 28, 44, and 68 hpi, MTT labeling reagent (0.5 mg/ml) were added to cells, followed by further incubation for 4 hrs at 37°C. MTT reaction was terminated by adding solubilization reagent. Cell

viabilities are shown as the percentage of OD values (570 nm) compared to the mean OD values of mock-infected samples at corresponding time points (Means ± standard deviations of four different wells). Statistically significant differences of RVax-1-infected cells from rMP-12-infected cells were tested by two-way ANOVA (*p<0.05, **p<0.01, F = 45.10, df = 10).

Supplementary Figure 2

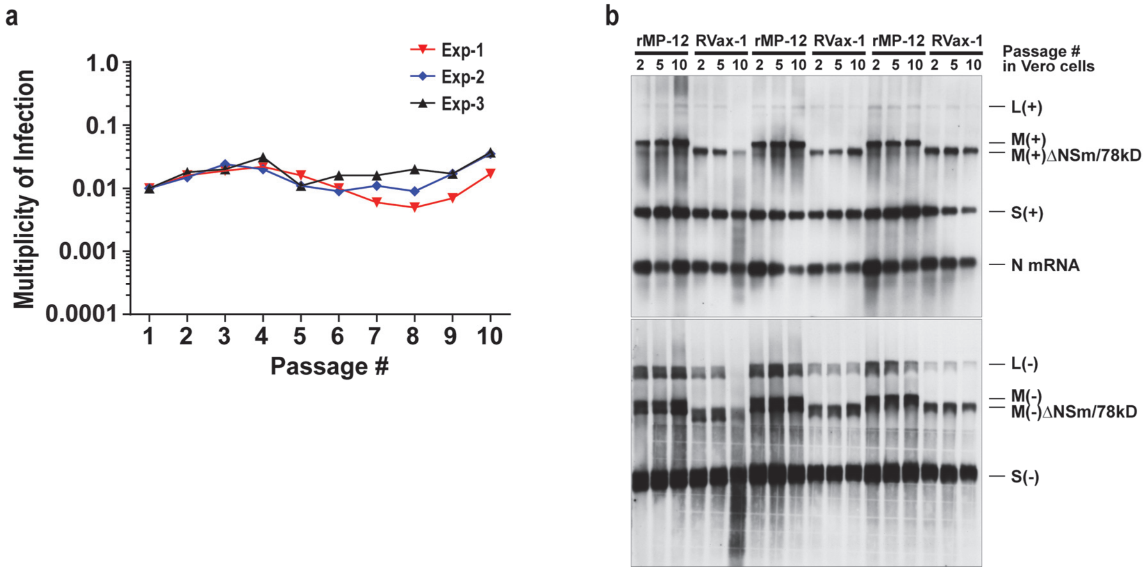

Supplementary Figure 2. Serial passages of RVax-1 in Vero cells. Vero cells were infected with rMP-12 or RVax-1 (P0 stock) at MOI 0.01. Ten serial passages of culture supernatants were performed with three independent series. **(a)** MOI at passage 1 to 10 of RVax-1 are shown. **(b)** Vero cells were infected with rMP-12 or RVax-1 (P1, 4, or 9 stock) at MOI 0.01, and total RNA samples were extracted at 48 hpi (P2, P5, or P10 samples). Total RNA was analyzed by Northern blot using a mixture of S, M, and L probes detecting positive-sense RNA (top panel) or negative-sense RNA (bottom panel). All blots were from the same experiment and processed in parallel.

Supplementary Figure 3

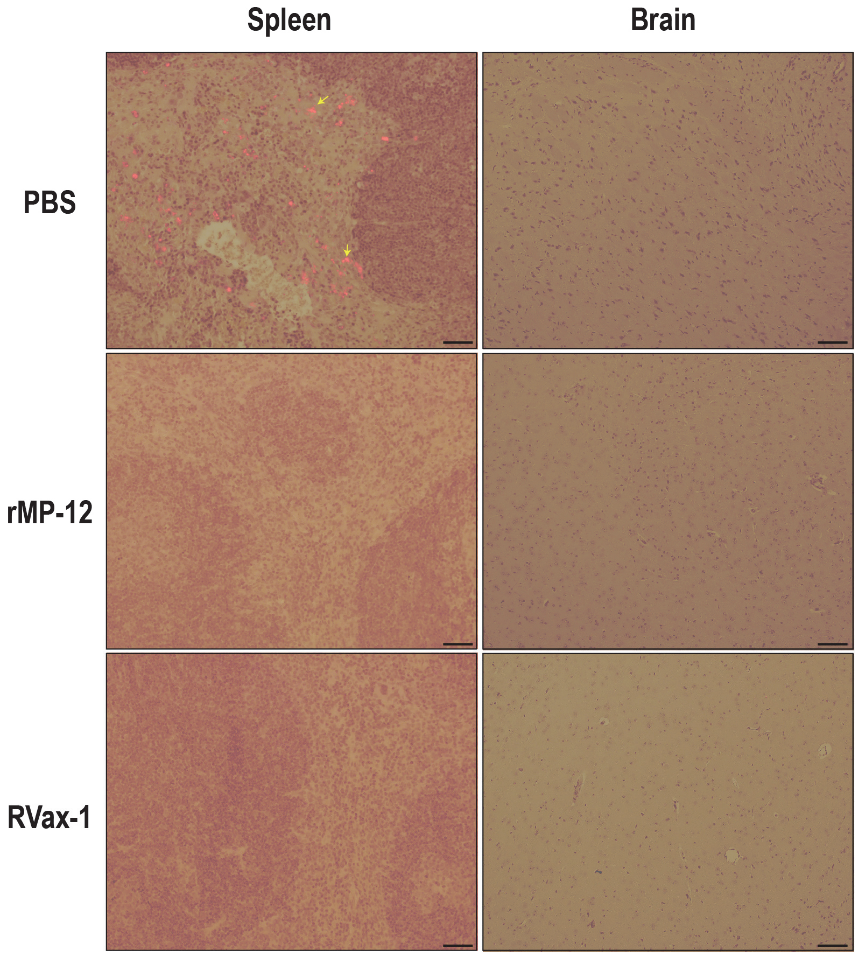

Supplementary Figure 3. Immunohistochemistry (IHC) for spleen and brain sections. IHC was performed using anti-RVFV N antibody for spleen (left panels) or brain sections (right panels) from rZH501-challenged, mock-vaccinated (PBS) or vaccinated (rMP-12 or RVax-1) mice. Images for brains represent an area of thalamus. Merged images under brightfield microscopy and TRITC fluorescent filter are shown. N antigen-positive cells are detected in red (arrows) in spleens but not in brains of mock-vaccinated animals. No RVFV N antigens were detected in vaccinated animals in spleens or brains. Bars represent 50 μm (left panels) or 100 μm (right panels).
