# Supplementary material for: Evaluations of rationally designed rift valley fever vaccine candidate RVax-1 in mosquito and rodent models
Source: NPJ Vaccines. 2022 Sep 21;7:109. doi: 10.1038/s41541-022-00536-3 (PMC9492667; doi:10.1038/s41541-022-00536-3)
Supplement: Supplementary file 2 — REPORTING SUMMARY [file 41541_2022_536_MOESM2_ESM.pdf]

nature portfolio

Corresponding author(s): Tetsuro Ikegami

Last updated by author(s): Aug 19, 2022

Reporting Summary

Nature Portfolio wishes to improve the reproducibility of the work that we publish. This form provides structure for consistency and transparency in reporting. For further information on Nature Portfolio policies, see our Editorial Policies and the Editorial Policy Checklist.

Statistics

For all statistical analyses, confirm that the following items are present in the figure legend, table legend, main text, or Methods section.

n/a	Confirmed	
☐	☒	The exact sample size (n) for each experimental group/condition, given as a discrete number and unit of measurement
☐	☒	A statement on whether measurements were taken from distinct samples or whether the same sample was measured repeatedly
☐	☒	The statistical test(s) used AND whether they are one- or two-sided<br>*Only common tests should be described solely by name; describe more complex techniques in the Methods section.*
☐	☒	A description of all covariates tested
☐	☒	A description of any assumptions or corrections, such as tests of normality and adjustment for multiple comparisons
☐	☒	A full description of the statistical parameters including central tendency (e.g. means) or other basic estimates (e.g. regression coefficient) AND variation (e.g. standard deviation) or associated estimates of uncertainty (e.g. confidence intervals)
☐	☒	For null hypothesis testing, the test statistic (e.g. F, t, r) with confidence intervals, effect sizes, degrees of freedom and P value noted<br>*Give P values as exact values whenever suitable.*
☒	☐	For Bayesian analysis, information on the choice of priors and Markov chain Monte Carlo settings
☒	☐	For hierarchical and complex designs, identification of the appropriate level for tests and full reporting of outcomes
☐	☒	Estimates of effect sizes (e.g. Cohen's d, Pearson's r), indicating how they were calculated

Our web collection on statistics for biologists contains articles on many of the points above.

Software and code

Policy information about availability of computer code

Data collection	Relative diameter lengths from 20 randomly selected plaques of rMP-12 or RVax-1 were measured by Image J software 1.53a (RRID:SCR_003070) with the Cell Counter Plugin.
Data analysis	Sample sizes were designed by G*Power 3.1.9.7. (Research Resource Identifiers: RRID:SCR_013726). All statistical analyses were performed with GraphPad Prism 8.4.3. (RRID:SCR_002798).

For manuscripts utilizing custom algorithms or software that are central to the research but not yet described in published literature, software must be made available to editors and reviewers. We strongly encourage code deposition in a community repository (e.g. GitHub). See the Nature Portfolio guidelines for submitting code & software for further information.

Data

Policy information about availability of data

All manuscripts must include a data availability statement. This statement should provide the following information, where applicable:

- Accession codes, unique identifiers, or web links for publicly available datasets
- A description of any restrictions on data availability
- For clinical datasets or third party data, please ensure that the statement adheres to our policy

The full genome sequence of RVax-1 vaccine candidate can be found in GenBank (accession numbers ON211620 – ON211622). The raw RNA-Seq data are available via the Sequence Read Archive (SRA) database (the BioProject accession number: PRJNA825509).

Human research participants

Policy information about studies involving human research participants and Sex and Gender in Research.

Reporting on sex and gender	N/A
Population characteristics	N/A
Recruitment	N/A
Ethics oversight	N/A

Note that full information on the approval of the study protocol must also be provided in the manuscript.

Field-specific reporting

Please select the one below that is the best fit for your research. If you are not sure, read the appropriate sections before making your selection.

☒ Life sciences ☐ Behavioural & social sciences ☐ Ecological, evolutionary & environmental sciences

For a reference copy of the document with all sections, see nature.com/documents/nr-reporting-summary-flat.pdf

Life sciences study design

All studies must disclose on these points even when the disclosure is negative.

Sample size	Sample sizes were designed by G*Power 3.1.9.7. (Research Resource Identifiers: RRID:SCR_013726)39 so that the described group and measurement values will provide at least 80% power (α = 0.05) to detect a large effect size (d = 0.80).
Data exclusions	No data were excluded from analysis except for the lack of samples due to unexpected animal death.
Replication	To further evaluate the immunogenicity of RVax-1, we repeated the experiment by including 14, 28. 42, and 98 dpv samples. At this time, two doses (low dose: 2.5 x 103; standard dose: 1.0 x 105) of RVax-1 and rMP-12 were tested (Figure 5a). Overall results in this repeated experiment supported consistent finding that RVax-1 vaccination can provide a protection from lethal RVFV challenge as did parental rMP-12 vaccination.
Randomization	Six-week-old C57BL/6 mice (Charles River) were vaccinated IM with PBS (mock), or 1x105 PFU of rMP-12 or RVax-1 (5 males and 5 females per group: not randomized). This experiment was repeated once with two different doses of rMP-12 or RVax-1 (2.5x103 PFU or 1x105 PFU: 5 males and 5 females per group: not randomized). Six-week-old C57BL/6 mice (Charles River, 3 males and 3 females per group: not randomized) were IM vaccinated with PBS (mock), rMP-12 (1.0x105 PFU) or RVax-1 (1.0x105 PFU).
Blinding	All animal experiments were performed in unblinded manner for the safe handling of infectious samples.

Reporting for specific materials, systems and methods

We require information from authors about some types of materials, experimental systems and methods used in many studies. Here, indicate whether each material, system or method listed is relevant to your study. If you are not sure if a list item applies to your research, read the appropriate section before selecting a response.

Materials & experimental systems

n/a	Involved in the study	
☐	☒	Antibodies
☐	☒	Eukaryotic cell lines
☒	☐	Palaeontology and archaeology
☐	☒	Animals and other organisms
☒	☐	Clinical data
☒	☐	Dual use research of concern

Methods

n/a	Involved in the study	
☒	☐	ChIP-seq
☒	☐	Flow cytometry
☒	☐	MRI-based neuroimaging

Antibodies

Antibodies used	*Describe all antibodies used in the study; as applicable, provide supplier name, catalog number, clone name, and lot number.*

Validation	*Describe the validation of each primary antibody for the species and application, noting any validation statements on the manufacturer's website, relevant citations, antibody profiles in online databases, or data provided in the manuscript.*

Eukaryotic cell lines

Policy information about cell lines and Sex and Gender in Research

Cell line source(s)	Vero cells (Chlorocebus sp., kidney epithelial cells, ATCC CCL-81) or MRC-5 cells (human lung diploid cells, ATCC CCL-171) were maintained in DMEM (Gibco, Thermo Fisher Scientific Inc., Waltham MA), containing 10% fetal bovine serum (FBS, HyClone, GE Healthcare, Chicago IL), penicillin (100 U/ml, Gibco), and streptomycin (100 µg/ml, Gibco), in a humidified cell culture incubator (5% CO2, 37°C). BHK-21 cells (Mesocricetus auratus, baby hamster kidney fibroblast cells, ATCC CCL-10) were maintained in minimum essential medium (MEM) alpha containing 10% FBS, penicillin (100 U/ml), and streptomycin (100 µg/ml) at 37°C with 5% CO2, while BHK cells that stably express T7 RNA polymerase (BHK/T7-9 cells) were maintained with hygromycin B (600 µg/ml). C6/36 cells (Ae. albopictus, ATCC CRL-1660) were maintained at 28ºC without CO2 in Leibovitz's L-15 medium containing 10% FBS, 10% tryptose phosphate broth (TPB), penicillin (100 U/ml), and streptomycin (100 µg/ml).
Authentication	The identities of MRC-5 cells were authenticated by Short Tandem Repeat analysis (UTMB Molecular Genomics Core Facility).
Mycoplasma contamination	Cells used in this study were verified to be mycoplasma free at the University of Texas Medical Branch at Galveston (UTMB) Tissue Culture Core Facility.
Commonly misidentified lines (See ICLAC register)	MRC-5 cells: commonly misidentified as HeLa cells (MRC-5 cells is a diploid cells used for vaccine study).

Animals and other research organisms

Policy information about studies involving animals; ARRIVE guidelines recommended for reporting animal research, and Sex and Gender in Research

Laboratory animals	Six-week-old C57BL/6 mice (Charles River) were vaccinated IM with PBS (mock), or 1x105 PFU of rMP-12 or RVax-1 (5 males and 5 females per group: not randomized). This experiment was repeated once with two different doses of rMP-12 or RVax-1 (2.5x103 PFU or 1x105 PFU: 5 males and 5 females per group: not randomized). Six-week-old C57BL/6 mice (Charles River, 3 males and 3 females per group: not randomized) were IM vaccinated with PBS (mock), rMP-12 (1.0x105 PFU) or RVax-1 (1.0x105 PFU).
Wild animals	N/A
Reporting on sex	Six-week-old C57BL/6 mice (Charles River) were vaccinated IM with PBS (mock), or 1x105 PFU of rMP-12 or RVax-1 (5 males and 5 females per group: not randomized). This experiment was repeated once with two different doses of rMP-12 or RVax-1 (2.5x103 PFU or 1x105 PFU: 5 males and 5 females per group: not randomized). Six-week-old C57BL/6 mice (Charles River, 3 males and 3 females per group: not randomized) were IM vaccinated with PBS (mock), rMP-12 (1.0x105 PFU) or RVax-1 (1.0x105 PFU).
Field-collected samples	N/A
Ethics oversight	All experiments using recombinant DNA and infectious RVFV have been performed upon the approval of the Notification of Use (#2021017 and #2019025) by the Institutional Biosafety Committee at UTMB. Mouse studies were performed in the UTMB Robert E. Shope or GNL BSL-4 laboratory accredited by the Association for Assessment and Accreditation of Laboratory Animal Care (AAALAC) in accordance with the Animal Welfare Act, NIH guidelines, and US federal law. Animal protocol #1912097 was approved by UTMB Institutional Animal Care and Use Committee (IACUC). All work with pathogenic rZH501 was performed in the Robert E. Shope or GNL BSL-4 laboratory, UTMB.

Note that full information on the approval of the study protocol must also be provided in the manuscript.
